# Store-operated Ca^2 +^ entry in proliferating and retinoic acid-differentiated N- and S-type neuroblastoma cells

**DOI:** 10.1016/j.bbamcr.2012.11.025

**Published:** 2013-03

**Authors:** Natalie Bell, Victoria Hann, Christopher P.F. Redfern, Timothy R. Cheek

**Affiliations:** a Institute for Cell and Molecular Biosciences, The Medical School, Newcastle University, Framlington Place, Newcastle upon Tyne, NE2 4HH, UK; b Northern Institute for Cancer Research, The Medical School, Newcastle University, Framlington Place, Newcastle upon Tyne, NE2 4HH, UK

**Keywords:** Store-operated Ca^2 +^ entry, Differentiation, STIM1, Orai1, TRPC1, Neuroblastoma

## Abstract

Neuroblastoma cell lines are heterogeneous, comprised of at least three distinct cell phenotypes; neuroblastic N-type cells, non-neuronal substrate-adherent S-type cells and intermediate I-type cells. N- and S-type cell populations were enriched from the parental SH-SY5Y neuroblastoma cell line and induced to differentiate by the addition of retinoic acid (RA), a drug used in the treatment of neuroblastoma. N- and S-type cells were identified based on their differential expression of β-tubulin III, vimentin and Bcl-2. Store-operated Ca^2 +^ entry (SOCE) was then measured in proliferating and differentiated N- and S-type cell populations and the expression of STIM1, Orai1 and TRPC1, three proteins reported to play a key role in SOCE, was determined. In N-type cells the RA-induced switch from proliferation to differentiation was accompanied by a down-regulation in SOCE. STIM1 and Orai1 expression became down-regulated in differentiated cells, consistent with their respective roles as ER Ca^2 +^ sensor and store-operated Ca^2 +^ channel (SOC). TRPC1 became up-regulated suggesting that TRPC1 is not involved in SOCE, at least in differentiated N-type cells. In S-type cells SOCE remained active following the RA-induced switch from proliferation to differentiation and the expression of STIM1 and Orai1 remained unchanged. TRPC1 was not expressed in S-type cells. Our results indicate that differentiation of neuronal cells is associated with a remodelling of SOCE. Therapeutic targeting of SOCE proteins could potentially be a means of promoting neuronal differentiation in the treatment of neuroblastoma.

## Highlights

► SOCE is down-regulated in differentiated N-type cells. ► STIM1 and Orai1 expression is down-regulated in differentiated N-type cells. ► TRPC1 expression is up-regulated in differentiated N-type cells. ► SOCE proteins could provide a therapeutic target in the treatment of neuroblastoma.

## 1 Introduction

Neuroblastoma is a childhood cancer of the sympathetic nervous system that originates from neural crest cells [1]. Neuroblastoma tumours and their derived cell lines are heterogeneous, composed of multipotent precursor cells that give rise to distinct neural crest cell lineages [2]. At least three cellular phenotypes have been identified in neuroblastoma cell lines; neuroblastic N-type cells, substrate-adherent S-type cells and intermediate I-type cells [2–5]. N-type cells are immature nerve cells, precursors to the sympathoadrenal cell lineage of the neural crest [4–6]. S-type cells are multipotent precursors to Schwann cells, melanocytes and glial cells and form the non-neuronal lineage of the neural crest [3,4]. I-type cells are intermediate with respect to N- and S-type cells in terms of morphology and biochemical markers [3,4]. I-type cells may represent either a stem cell or an intermediate stage in the transdifferentiation between N- and S-type cells [3,7]. N-type cells are more malignant than S-type cells, which appear to be non-malignant [5,8,9]. Retinoic acid (RA) is used in the treatment protocol for high-risk neuroblastoma patients as it inhibits proliferation and induces differentiation of cells [10–12].

The second messenger Ca^2 +^ plays an essential role in the regulation of many cellular processes [13,14], including differentiation of neuronal cells [15,16]. Store-operated Ca^2 +^ entry (SOCE) is a ubiquitous Ca^2 +^ influx pathway through which Ca^2 +^ enters cells via store-operated Ca^2 +^ channels (SOCs) located in the plasma membrane (PM) in response to depletion of endoplasmic reticulum (ER) Ca^2 +^ stores [17,18]. Previous work from this laboratory has shown that SOCE becomes down-regulated in SH-SY5Y neuroblastoma cells following 9-*cis* retinoic acid (9*c*RA)-induced differentiation [19].

The proteins STIM1, Orai1 and TRPC1 have been reported to play a key role in SOCE [20–23]. STIM1 senses the level of Ca^2 +^ within the ER and re-locates to ER-PM junctions to signal store depletion and induce opening of SOCs [24,25]. Orai1 forms a SOC in many cell types and is required to reconstitute the Ca^2 +^ release-activated Ca^2 +^ current (I_CRAC_) [21,26], the most well-defined SOCE pathway. TRPC1 is a controversial SOC candidate as literature both supports and opposes the involvement of TRPC1 in SOCE [18,27]. TRPC1 may only function as a SOC under certain conditions as studies have shown that TRPC1 can function as either a SOC or a receptor-operated channel (ROC) depending on its interaction with STIM1 [28–30]. The interaction between STIM1 and TRPC1 can also require Orai1 [29,31–34]. Accumulating evidence suggests that SOCs are heteromeric complexes that can include both Orai1 and TRPC1 [29,31,33,34].

In the present study, N- and S-type cells were enriched from the parental SH-SY5Y neuroblastoma cell line which, although mainly composed of N-type cells, S-type cells remain present due to the ability of cells to transdifferentiate between cell phenotypes [7,35]. Cell populations were induced to differentiate by the addition of 9*c*RA and characterised morphologically and biochemically using the neuronal marker proteins β-tubulin III and Bcl-2 [36–39] and the non-neuronal marker protein vimentin [3]. The remodelling of SOCE observed following 9*c*RA-induced differentiation [19] was further characterised in this study by determining the extent that N- and S-type cells contribute to the down-regulation. The pattern of expression of STIM1, Orai1 and TRPC1 was also determined in proliferating and differentiated N- and S-type cells to investigate the involvement of these Ca^2 +^ signalling proteins in the remodelling of SOCE.

## 2 Materials and methods

### 2.1 Materials

SH-SY5Y cells were supplied by R. Ross (Fordham University, NY, USA). FluorSave, fura-2/AM, ionomycin and thapsigargin (TG) were obtained from Calbiochem (Darmstadt, Germany). All other chemicals were obtained from Sigma-Aldrich (Dorset, UK) unless otherwise stated.

### 2.2 Cell culture and differentiation

SH-SY5Y, N- and S-type neuroblastoma cells were cultured in Dulbecco's modified Eagle's medium (DMEM)/F12:1 with GlutaMAX™ (Gibco, Paisley, UK) supplemented with foetal calf serum (10%), penicillin (100 IU. ml^− 1^) and streptomycin (100 IU^.^ml^− 1^). Cells were kept at 37 °C in a humidified atmosphere of 5% CO_2_. SH-SY5Y cells were passaged once a week using 0.02% EDTA and were not used beyond passage 28. Cells were seeded onto coverslips/dishes at least 24 h prior to the start of treatment. For differentiation, cells were treated for 7 days with 1 μM 9*c*RA. Differentiation medium was replaced every 2 days. Proliferating (control) cells were treated identically but with an equal volume of vehicle ethanol (0.01%) in place of 9*c*RA.

### 2.3 Enrichment for N- and S-type cells

N- and S-type cells were enriched from the parental SH-SY5Y neuroblastoma cell line on the basis of their differential substrate adherence [8]. N-type cell populations were obtained by knocking off the more weakly adherent cells into PBS by gentle agitation and transferring the cell suspension to a new flask; S-type cell populations were obtained by maintaining those still adhered to the flask. N- and S-type cell populations were sub-cultured in this way 8 times and are referred to in the text as N- and S-type cells.

### 2.4 Immunofluorescence

SH-SY5Y, N- and S-type neuroblastoma cells were fixed with 4% paraformaldehyde and permeabilised with 0.1% Triton X-100. Cells were blocked with 5% bovine serum albumin (BSA) prior to incubation for 2 h at 4 °C with anti-β-tubulin III with Alexa Fluor 488 conjugate, 1:50 (Covance, NJ, USA ) and anti-vimentin with Alexa Fluor 674 conjugate, 1:50 (Santa Cruz, CA, USA). Cells were incubated with ethidium homodimer-1 (EthD-1) at a 1:500 dilution for 10 min at room temperature (RT). Coverslips were mounted onto glass slides with FluorSave. Images were collected on a Zeiss LSM 510 confocal microscope using a 40 × oil immersion objective. A multi-track configuration was used with 488 nm and 633 nm excitation light from an argon laser and a helium-neon laser respectively. Confocal images were collected with the pinhole set at 1 Airy unit for Alexa Fluor 647 emission, the same optical slice (1.1 μm) was then set for the collection of Alexa Fluor 488 and EthD-1 emission.

### 2.5 Western blotting

Cells were lysed with 4 °C buffer containing 1 mM EDTA (pH 8), 1 mM EGTA (pH 8), 1.28 mM sucrose, 2 mM Tris (pH 7.6), 10% Triton X-100 and protease inhibitor cocktail (Roche, Hertfordshire, UK). Cells were scraped off dishes, passed through a 20-gauge needle several times and centrifuged at 10,000 rpm for 10 min at 4 °C. Protein concentration was measured at 492 nm using the Coomassie Blue Reagent (BioRad, Hertfordshire, UK). BSA was used as a protein standard to determine the protein concentration of samples.

SDS-PAGE was performed using the NuPAGE system (Invitrogen, Paisley, UK). Total protein (20–40 μg) was separated by electrophoresis through 10% Bis-Tris gels (Invitrogen) and transferred onto nitrocellulose membranes (BioRad). Blots were blocked for 1 h (5% milk, 0.02% Triton X-100) and were incubated with primary antibody in incubation buffer (2.5% milk) overnight at 4 °C. Primary antibodies; anti-β-actin 1:10,000 (abcam, Cambridge, UK), anti-β-Tubulin III, 1:20,000 (Covance), anti-vimentin, 1:200, (Santa Cruz), anti-Bcl-2, 1:200 (Santa Cruz), anti-STIM1, 1:200 (BD Biosciences, NJ, USA), anti-Orai1, 1:100 and anti-TRPC1, 1:200 (Alamone Labs, Jerusalem, Israel). Blots were then incubated with either mouse or rabbit secondary antibody with horseradish peroxidase-conjugate (Dako, Glostrup, Denmark) for 1 h at RT. The enhanced chemiluminescence system was used to detect immunoreactive bands on Hyperfilm™ (GE Healthcare, Buckinghamshire, UK). Band intensities were measured using ImageJ® software. Protein levels were normalised to the levels of β-actin.

### 2.6 Determination of [Ca^2 +^]_i_ in cell populations

SH-SY5Y, N- and S-type cells were washed in Krebs buffer (10 mM glucose, 118 mM NaCl, 4.7 mM KCl, 1.2 mM KH_2_PO_4_, 1.2 mM MgSO_4_, 4.2 mM NaHCO_3_, 2 mM CaCl_2_ and 200 μM sulfinpyrazone, 10 mM HEPES, pH 7.4) and loaded with the Ca^2 +^-sensitive fluorescent indicator dye fura-2/AM (3 μM) for 45 min. Following loading cells were washed in Krebs buffer and incubated for a further 20 min to allow de-esterification of the loaded dye. Cells were washed in Ca^2 +^-free Krebs buffer before being mounted into a coverslip holder (PerkinElmer, Beaconsfield, UK) and inserted into a stirred cuvette containing Ca^2 +^-free Krebs buffer. Before beginning each experiment, an excitation wavelength scan (250–450 nm) was performed to reveal any large inconsistency in the degree of confluency and/or dye loading. Less than 5% of coverslips were rejected on this basis. Fura-2 fluorescence was continuously monitored using a PerkinElmer LS-50B fluorimeter, with excitation and emission wavelengths of 340 and 510 nm respectively. Following the establishment of a steady baseline TG (200 nM) was added to deplete ER Ca^2 +^ stores. CaCl_2_ (2 mM) was then added back to the Ca^2 +^-free Krebs buffer to reveal subsequent Ca^2 +^ entry. Ionomycin (50 μM) was added to obtain *F*_max_ (maximum fura-2 fluorescence) and MnCl_2_ (1 mM) was added to obtain *F*_Mn_ (fluorescence after quenching of fura-2), an indirect measurement of *F*_min_ (minimum fura-2 fluorescence). [Ca^2 +^]_i_ was calculated using PerkinElmerWinLab® software which uses the formula of Grynkiewicz [40] which assumes a dissociation constant (*K_d_*) of 224 nm and an instrument constant (IC) of 3.The following equations were used; *F*_min_ = 1 / IC (*F*_max_ − *F*_Mn_) + *F*_Mn_ and [Ca^2 +^]_i_ = *K*_d_(*F* − *F*_min_) / (*F*_max_ − *F*) [40]. Each trace was calibrated individually to account for variation in confluency and/or fura-2 loading. To quantify change in [Ca^2 +^]_i_ the area from each response was determined in calibrated traces using PerkinElmerWinLab® software. The area from calibrated DMSO control traces was subtracted from experimental (TG) traces.

### 2.7 Statistical analysis

Data are presented as means ± SEM of at least three independent experiments, as stated in the Results section. Comparisons between unpaired groups were carried out using two-tailed Student's t-tests in GraphPad Prism® software. Data presented in Table 1 were analysed using 2-way ANOVA (after log-transformation) and general linear models in R [41]. For all tests, the criterion for statistical significance was *P* < 0.05.

## 3 Results

### 3.1 Enrichment for N- and S-type cells

**Fig. 1:**
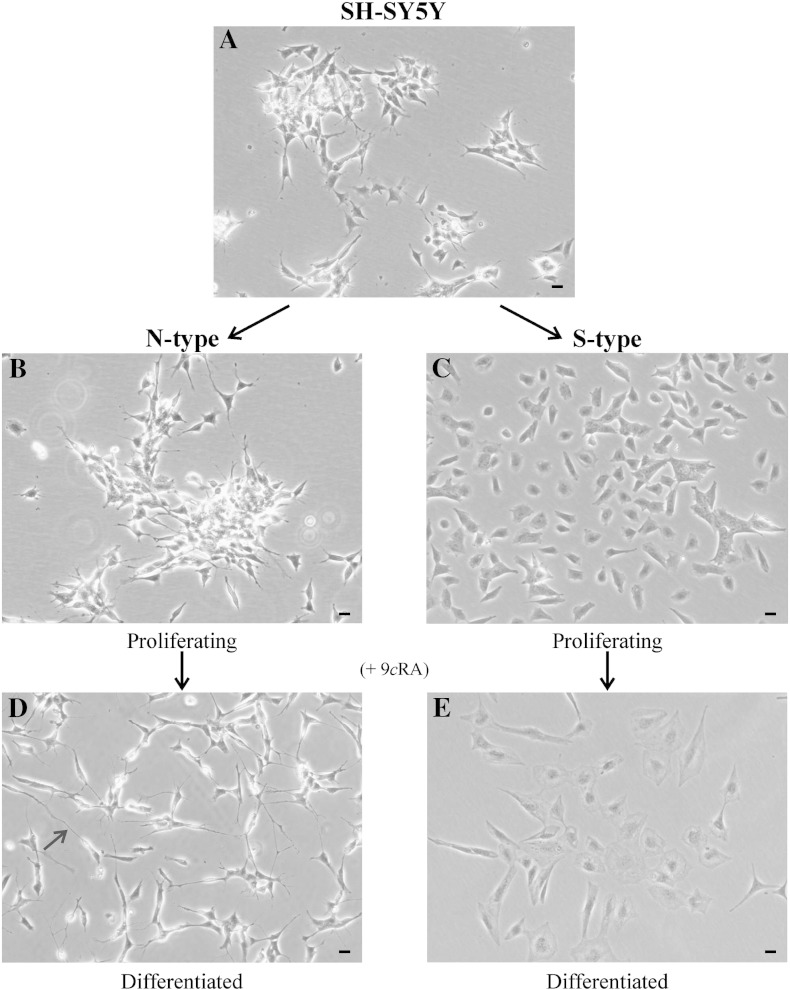
N- and S-type cell populations were enriched from the parental SH-SY5Y neuroblastoma cell line and induced to differentiate by the addition of 1 μM 9*c*RA for 7 days. (A) The SH-SY5Y cell line was predominantly composed of N-type cells though S-type cells were present. Proliferating (B) N-type and (C) S-type cells. (D) Differentiated N-type cells. Cells exhibited neurite extensions of ≥ 50 μm in length; arrow. (E) Differentiated S-type cells. Scale bars represent 20 μm.

The SH-SY5Y cell line was predominantly composed of N-type cells (Fig. 1A). However, S-type cells were present (Fig. 1A), and in our laboratory comprised approximately 20% of the total cell population. For our studies N- and S-type cells were enriched from the parental SH-SY5Y cell line (Fig. 1). In N-type cell populations S-type cells comprised less than 3% of the total cell population, similarly in S-type cells populations N-type cells comprised less than 3% of the total cell population. Proliferating N-type cells were small with rounded or slightly elongated cell bodies with several short, branched neurite-like processes (Fig. 1B). N-type cells grew rapidly with a doubling time of ~ 24 h and formed weakly adherent cellular aggregates. Proliferating S-type cells were larger and flatter than N-type cells and exhibited strong substrate adherence (Fig. 1C). S-type cells grew more slowly compared to N-type cells with a doubling time of ~ 48 h and showed contact inhibition of growth.

### 3.2 Differentiation of N- and S-type cells

After treatment with 9*c*RA, N-type cells differentiated into a neuronal-like phenotype, exhibiting long neurite extensions (Fig. 1D, arrow). Cells were classed as differentiated if neurite extensions were ≥ 50 μm in length [19,42,43]. S-type cells differentiated into a more epithelial-like phenotype becoming larger and flatter with increased substrate adherence (Fig. 1E). RA treatment inhibits proliferation of cells [10] and a reduction in the rate of proliferation by ~ 75% in N-type cells and by ~ 50% in S-type cells was observed.

### 3.3 Immunofluorescent profile of N- and S-type cells

**Fig. 2:**
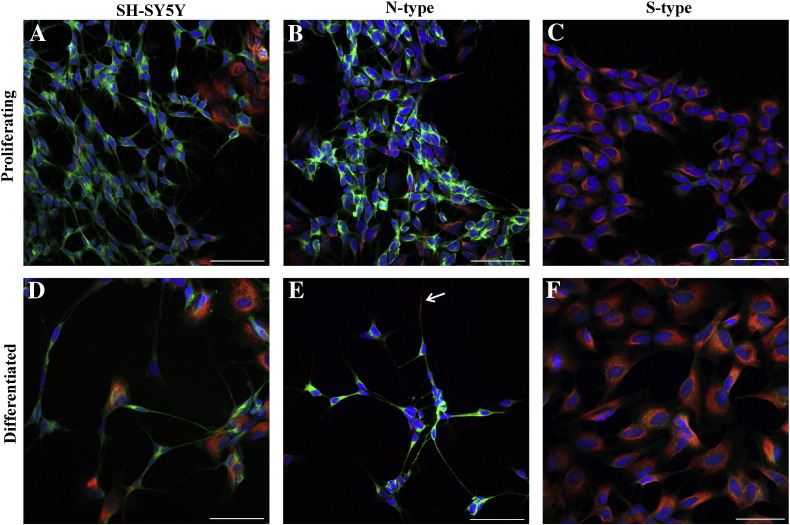
N-type cells stained positive for a neuronal lineage marker whereas S-type cells stained positive for a non-neuronal lineage marker. Confocal micrographs showing β-tubulin III (green), vimentin (red) and EthD-1 (blue). Cells were induced to differentiate by the addition of 1 μM 9*c*RA for 7 days. Proliferating (A) SH-SY5Y, (B) N-type and (C) S-type cells. Differentiated (D) SH-SY5Y and (E) N-type cells. Vimentin in neurite extension; arrow. (F) Differentiated S-type cells. Scale bars represent 50 μm. The parental SH-SY5Y cell line was comparable to N-type cells.

In order to determine cell lineage, fixed cells were stained with antibodies directed against β-tubulin III, a component of microtubules in neuronal cells [38,39] and vimentin, an intermediate filament protein in non-neuronal cells [3]. β-tubulin III was present throughout the cytoplasm and along neurite branches/extensions of both proliferating and differentiated N-type cells; expression did not appear to change after 9*c*RA-induced differentiation (Fig. 2B, E). β-tubulin III was also present throughout the cytoplasm of S-type cells, albeit at a weaker level compared to N-type cells (Fig. 2C, F). Vimentin was not generally present in the cell cytoplasm of N-type cells, though it was identified in some neurite extensions (Fig. 2E, arrow). Vimentin was present in the cytoplasm of both proliferating and differentiated S-type cells (Fig. 2C, F). In the parental SH-SY5Y cell line, N-type cells formed the majority of the population but S-type cells remained present (Fig. 2A, D).

### 3.4 β-tubulin III, vimentin and Bcl-2 in N- and S-type cells

**Fig. 3:**
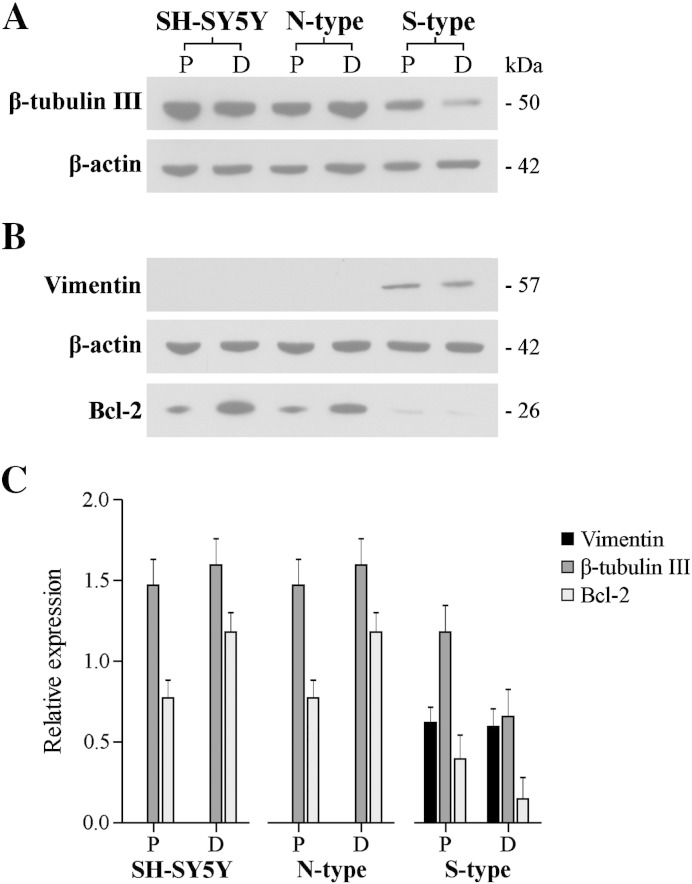
N- and S-type cells show differential expression of β-tubulin III, vimentin and Bcl-2. P = proliferating (vehicle EtOH), D = differentiated (1 μM 9*c*RA). β-actin was used as a loading control. (A) Western blot showing expression of β-tubulin III. In differentiated S-type cells expression became down-regulated compared to proliferating S-type cells. (B) Western blots showing expression of vimentin and Bcl-2. Vimentin was only detected in S-type cells. Bcl-2 expression became up-regulated in differentiated SH-SY5Y and N-type cells and was barely detectable in S-type cells. (C) Histogram showing relative expression of β-tubulin III, vimentin and Bcl-2 as a ratio of β-actin expression. The parental SH-SY5Y cell line was comparable to N-type cells.

The expression of β-tubulin III and vimentin in N- and S-type cells was quantified by western blot analysis. In N-type cells β-tubulin III levels remained unchanged after 9*c*RA-induced differentiation, *P* = 0.118, *n* = 9 (Fig. 3A,C). In S-type cells β-tubulin III was present, though at a lower level compared to the N-type cells, and after 9*c*RA treatment became down-regulated compared to proliferating S-type cells by ~ 44%, *P* < 0.001, *n* = 9 (Fig. 3A, C). The expression of vimentin in S-type cells remained unchanged following 9*c*RA-induced differentiation, *P* = 0.879, *n* = 8 (Fig. 3B, C). Although vimentin was identified in some neurite extensions of N-type cells (Fig. 2E), it was not detected by western blot (Fig. 3B). The parental SH-SY5Y cell line was comparable to N-type cells (Fig. 3).

The expression of Bcl-2 was also determined as Bcl-2 has been shown to increase in differentiated neuroblastoma cells [44–46]. Bcl-2 was expressed in proliferating SH-SY5Y and N-type cells and after 9*c*RA-induced differentiation became up-regulated by ~ 50% (*P* = 0.020, *n* = 6) and ~ 35% (*P* = 0.027, *n* = 7) respectively (Fig. 3B, C). Bcl-2 expression was barely detectable in S-type cells (Fig. 3B, C).

### 3.5 SOCE in N- and S-type cells

Ca^2 +^ ‘add-back’ experiments were performed on N- and S-type cell populations to determine any changes in SOCE that may have occurred in response to 9*c*RA-induced differentiation. Fura-2 loaded cells in Ca^2 +^-free buffer were stimulated with TG (200 nM) to deplete ER Ca^2 +^ stores, and then CaCl_2_ (2 mM) was added to determine subsequent Ca^2 +^ entry (i.e. SOCE).

**Fig. 4:**
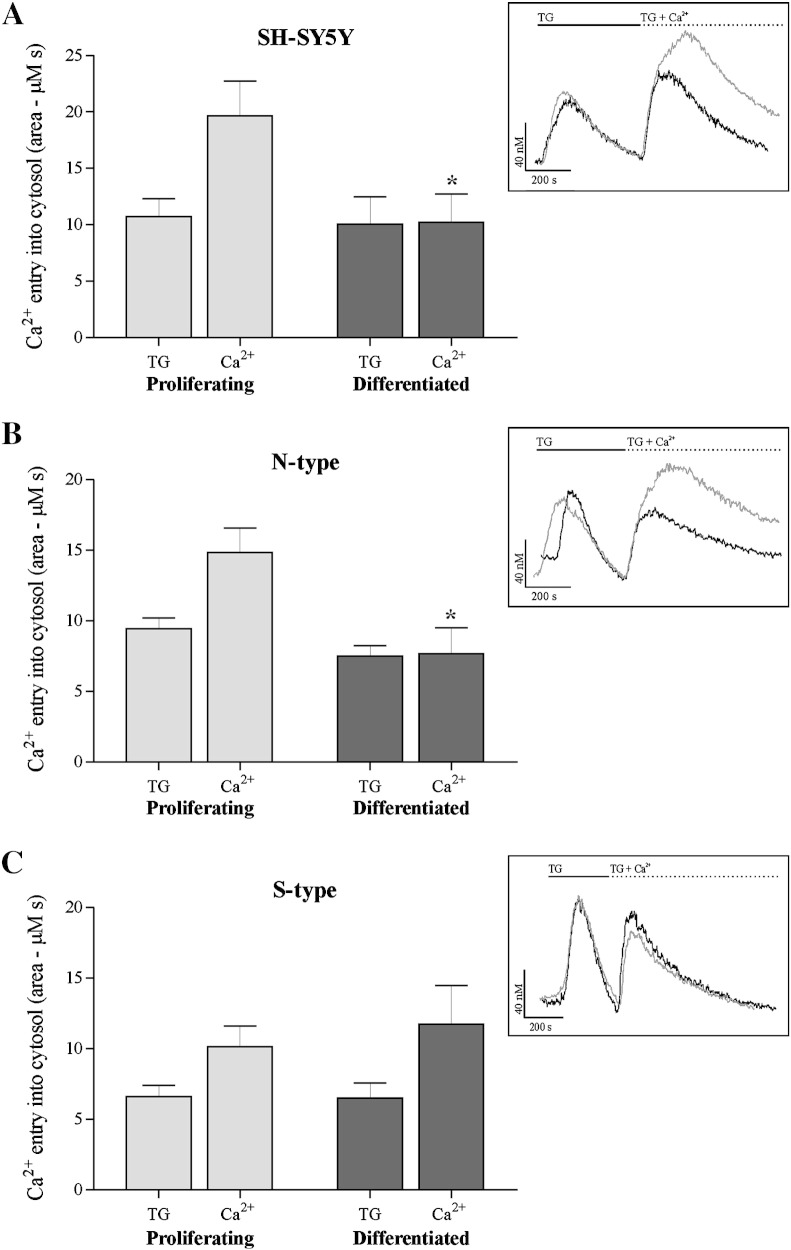
SOCE pathway activity in proliferating and 9*c*RA differentiated N- and S-type cells. Cells were induced to differentiate by the addition of 1 μM 9*c*RA for 7 days. The addition of TG (200 nM) to fura-2 loaded cells depletes ER Ca^2 +^ stores which is observed as an increase in [Ca^2 +^]_i_ and the re-addition of extracellular Ca^2 +^ (CaCl_2_ , 2 mM) to Ca^2 +^-free buffer is also observed as an increase in [Ca^2 +^]_i_ as Ca^2 +^ enters the cells (calibrated traces show [Ca^2 +^]_i_ (40 nM) against time (200 s)); () proliferating, (■) differentiated. After 9*c*RA-induced differentiation, SOCE became in (A) SH-SY5Y cells, significantly down-regulated (*), in (B) N-type cells, significantly down-regulated (*) and in (C) S-type cells, unchanged.

In N-type cells the extent of depletion from ER Ca^2 +^ stores in response to the addition of TG was not significantly different between proliferating and differentiated cells; 9.45 ± 0.78 μM s (*n* = 24) vs. 7.50 ± 0.76 μM s (*n* = 18), *P* = 0.088 (Fig. 4B). However, subsequent Ca^2 +^ entry following add-back of Ca^2 +^ was significantly reduced by ~ 48% in differentiated cells compared to proliferating cells; 14.85 ± 1.74 μM s vs. 7.64 ± 1.83 μM s, *P* = 0.002 (Fig. 4B). The SOCE pathway therefore becomes down-regulated in differentiated N-type cells. This result is comparable to that seen in the parental SH-SY5Y cell line (Fig. 4A), and also that previously observed in this laboratory [19].

In S-type cells the extent of depletion from ER Ca^2 +^ stores in response to TG was not significantly different between proliferating and differentiated cells; 6.63 ± 0.77 μM s (*n* = 15) vs. 6.50 ± 1.10 μM s (*n* = 16), *P* = 0.925 (Fig. 4C). Subsequent Ca^2 +^ entry following add-back of Ca^2 +^ was also not significantly different between proliferating and differentiated cells; 10.15 ± 1.48 μM s vs. 11.74 ± 3.14 μM s, *P* = 0.657 (Fig. 4C). The SOCE pathway therefore remained active in differentiated S-type cells.

**Fig. 5:**
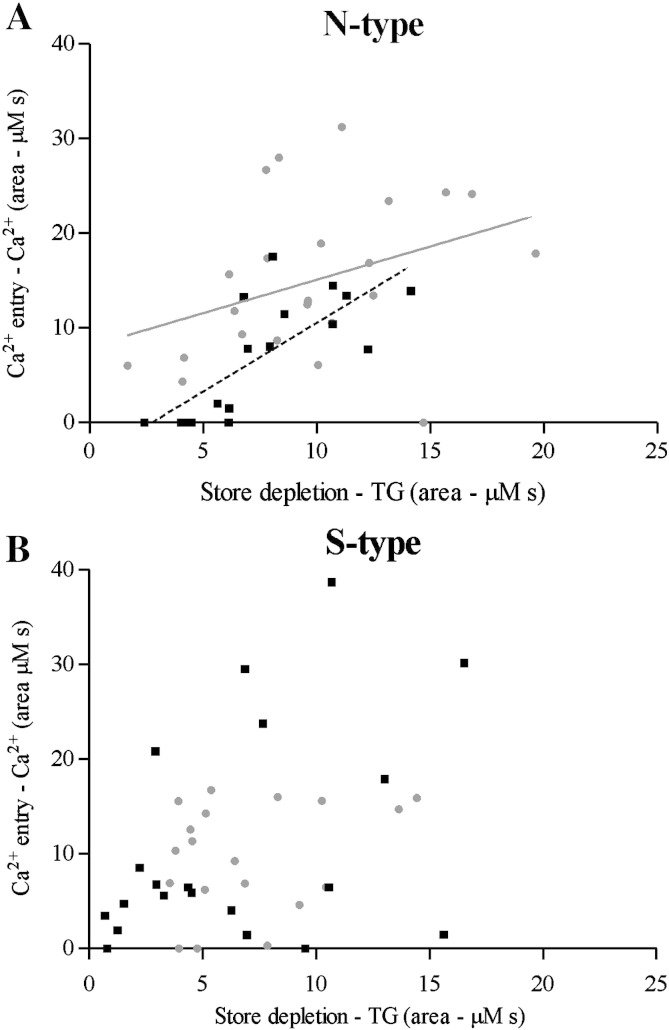
Relationship between store depletion and subsequent Ca^2 +^ entry in () proliferating and (■) differentiated N- and S-type cell populations. (A) For N-type cells the overall model was statistically significant (*P* < 0.001). (B) For S-type cells the overall model was not significant, *P* = 0.262. Analysis was in R.

To understand further the relationship between store depletion and subsequent Ca^2 +^ entry during the switch from proliferation to differentiation, TG responses (store depletion) were plotted against Ca^2 +^ responses for individual N- and S-type cell populations (Fig. 5) and analysed using linear models to investigate the effect of TG response and 9*c*RA treatment on subsequent Ca^2 +^ entry. For N-type cells there was a significant effect of store depletion on Ca^2 +^ entry (*P* < 0.001) and a significant negative effect of 9*c*RA treatment (i.e. reduction in the intercept of the line relating TG to Ca^2 +^ response; *P* = 0.011) consistent with a down-regulation of Ca^2 +^ entry in differentiated cells (Fig. 5A). The slopes of the lines relating the magnitudes of store depletion to Ca^2 +^ entry did not differ significantly in relation to 9*c*RA treatment (*P* = 0.222). For S-type cells, there was no statistically-significant effect of store depletion or 9*c*RA treatment on Ca^2 +^ entry (model overall, *P* = 0.262; TG effect, *P* = 0.089; 9*c*RA effect and interaction *P* = 0.338) (Fig. 5C).

The rates of rise of both TG and Ca^2 +^ responses were also determined. There was a significant difference in the rates of rise for store depletion (*P* = 0.015) and Ca^2 +^ entry (*P* < 0.001) between N- and S-type cells (Table 1). However, the rates of rise for store depletion and Ca^2 +^ entry did not change significantly in response to 9*c*RA treatment (store depletion, *P* = 0.052; Ca^2 +^ entry, *P* = 0.944) regardless of cell type (2-way ANOVA interaction terms, *P* = 0.175) (Table 1). Although the rates of rise for store depletion did not strictly meet the criteria for statistical significance, the rather low probability of no effect in this case raises the possibility that 9*c*RA treatment may increase sensitivity to TG in both cell types.

### 3.6 STIM1, Orai1 and TRPC1 in N- and S-type cells

**Fig. 6:**
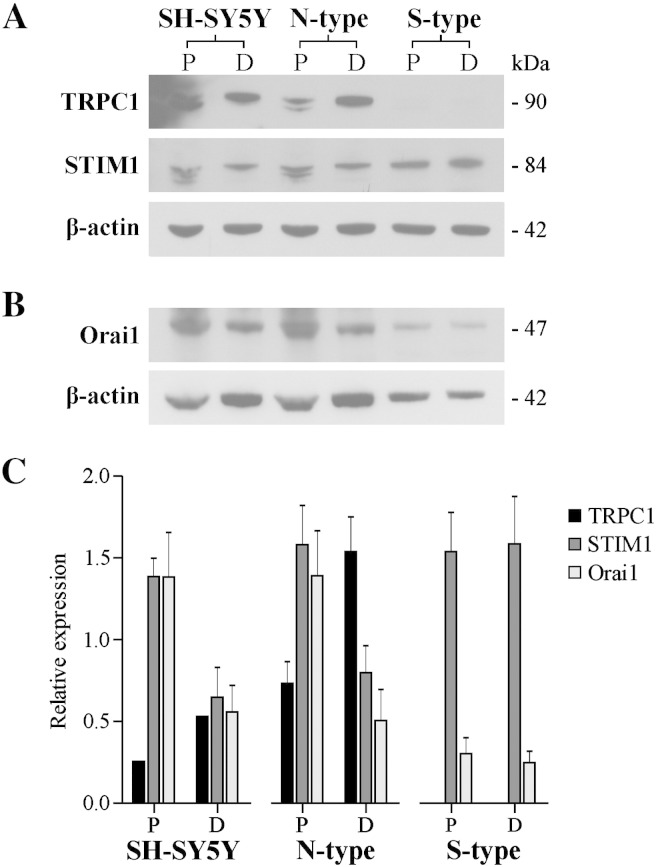
N- and S-type cells show differential expression of STIM1, Orai1 and TRPC1. P = proliferating (vehicle EtOH), D = differentiated (1 μM 9*c*RA). β-actin was used as a loading control. (A) Western blots showing expression of TRPC1 and STIM1. TRPC1 expression became up-regulated in N-type after differentiation and was not expressed in S-type cells. STIM1 expression became down-regulated in SH-SY5Y and N-type cells after 9*c*RA-induced differentiation and remained unchanged in S-type cells. (B) Western blot showing expression of Orai1. Orai1 expression became down-regulated in SH-SY5Y and N-type cells after 9*c*RA-induced differentiation and remained unchanged in S-type cells. (C) Histogram showing relative expression of β-tubulin III, vimentin and Bcl-2 as a ratio of β-actin expression. The parental SH-SY5Y cell line was comparable to N-type cells.

The proteins STIM1, Orai1 and TRPC1 have previously been shown to play a key role in SOCE [20–23]. To determine whether these proteins were involved in the SOCE activity measured in N- and S-type cells, their expression was measured by western blot analysis. STIM1 was identified in both N- and S-type cells. After 9*c*RA-induced differentiation, the level of STIM1 expression became down-regulated by ~ 49%, *P* = 0.036 in N-type cells, though remained unchanged in S-type cells, *P* = 0.905, *n* = 4 (Fig. 6A,C). These changes were consistent with the changes observed in SOCE (Fig. 4). Orai1 was also identified in both N- and S-type cells. Following 9*c*RA-induced differentiation the level of Orai1 expression became down-regulated by ~ 64%, *P* = 0.038 in N-type cells, and remained unchanged in S-type cells, *P* = 0.661, *n* = 4 (Fig. 6B, C). The changes observed in Orai1 expression are consistent both with the changes observed in STIM1 expression (Fig. 6A, C), and also the level of SOCE activity measured in SH-SY5Y, N- and S-type cells (Fig. 4). TRPC1 was identified in N-type cells (Fig. 6A, C). After 9*c*RA-induced differentiation TRPC1 expression became up-regulated by ~ 52% (*P* = 0.032, *n* = 3) in N-type cells, suggesting that TRPC1 does not form a SOC in these cells since SOCE becomes down-regulated (Fig. 4B). Though proliferating and differentiated S-type cells display SOCE (Fig. 4C), TRPC1 was not identified in S-type cells (Fig. 6A, C). In proliferating N-type cells, a double band was observed in response to STIM1 and TRPC1 antibodies that was not observed in differentiated N-type cells (Fig. 6A). The parental SH-SY5Y cell line was comparable to the N-type cells (Fig. 6A, B).

## 4 Discussion

Previous findings from this laboratory have shown that SOCE becomes down-regulated in 9*c*RA-differentiated SH-SY5Y neuroblastoma cells [19]. The aims of this study were to characterise SOCE in N- and S-type cell populations and to investigate the involvement of STIM1, Orai1 and TRPC1 in relation to SOCE activity. N- and S-type cells were enriched from the parental SH-SY5Y cell line and the expression of proteins previously shown to be specific to N and S cell lines was determined to confirm neuronal and non-neuronal lineages respectively.

Differentiation of N- and S-type cells with 9*c*RA highlighted morphological differences between the two cell types with N-type cells becoming more neuronal-like and S-type cells becoming more epithelial-like.

β-tubulin III, a microtubule protein found in neuronal cells [38,39], was expressed in N-type cells, and expression did not change following 9*c*RA-induced differentiation. β-tubulin III therefore appears to be a marker of neuronal lineage and not of differentiation itself. β-tubulin III was also, surprisingly, expressed in S-type cells. Expression was weak as judged by immunofluorescence but clearly present as determined by western blot. S-type cells may have only recently committed to a non-neuronal lineage through the process of transdifferentiation [3,7]. Interestingly, β-tubulin III expression became further down-regulated in differentiated S-type cells. This is consistent with S-type cells moving away from a neuronal phenotype; it has been found that after RA treatment S-type cells differentiate into Schwann cells [47] and also melanocytic cells [48].

Vimentin, an intermediate filament protein found in non-neuronal cells [3], was expressed in S-type cells. Vimentin was not generally expressed in N-type cells but it was identified in some neurite extensions. Vimentin is, however, an essential transient requirement for the initiation of neurite outgrowth in NB2a neuroblastoma cells and also in hippocampal neurons where knockdown of vimentin significantly inhibited neurite outgrowth [49,50].

The anti-apoptotic protein Bcl-2, which is widely expressed in sympathetic neurons [37], was present in proliferating N-type cells and became up-regulated in differentiated N-type cells [44–46], consistent with the neuronal lineage of the N-type cells. Bcl-2 expression was barely detectable in both proliferating and differentiated S-type cells.

The differential expression of β-tubulin III, vimentin and Bcl-2 observed confirmed the morphological and biochemical enrichment of N- and S-type cells from the parental SH-SY5Y cell line. After enrichment, SOCE was measured in N- and S-type cell populations. The results revealed that the down-regulation of SOCE previously observed in SH-SY5Y cells [19], and also in this study, is a feature of N-type cells and not S-type cells.

The activity of the SOCE pathway was consistent with the level of expression of STIM1 and Orai1 in both cell types. In N-type cells SOCE became down-regulated following 9*c*RA-induced differentiation, as did the expression of STIM1 and Orai1. In S-type cells SOCE remained unchanged following 9*c*RA-induced differentiation, as did the expression of STIM1 and Orai1. This is consistent with STIM1 as the ER Ca^2 +^ sensor [20,22] in both N- and S-type cells and with Orai1 forming, at least in part, the SOC [21,22] in both N- and S-type cells. TRPC1 was present in proliferating N-type cells and expression became up-regulated following differentiation. TRPC1 has been found to function as a SOC when associated with STIM1 [28,30,31] and may therefore function as a SOC in proliferating N-type cells, however this was not determined. It is unlikely that TRPC1 functions as a SOC in differentiated N-type cells as TRPC1 expression became up-regulated whereas SOCE became down-regulated. In HEK293T cells down-regulation of STIM1 allowed TRPC1 to function as a ROC, insensitive to store depletion [30]. If TRPC1 functions as a ROC in differentiated N-type cells this could explain our previous observation that in 9*c*RA-differentiated SH-SY5Y cells a non-SOCE pathway becomes up-regulated [19]. TRPC1 may be associated with differentiation itself; TRPC1 was required for neurite outgrowth in differentiating PC12 cells but was independent of SOCE [51]. TRPC1 expression was not detectable in either proliferating or differentiated S-type cells, which would indicate that TRPC1 does not function as a SOC in S-type cells. An increased expression of Bcl-2 has been associated with an inhibition of SOCE [52,53] and may therefore also play a role in SOCE down-regulation in N-type cells.

Proliferating N- and S-type cells had similar SOCE profiles yet clearly the composition of proteins varies between cell types as Orai1 expression was generally lower in S-type cells and TRPC1 was not present. Differences in protein composition were further observed between differentiated N- and S-type cells as the expression of STIM1, Orai1 and TRPC1 changed in N-type cells. SOC composition could help explain the different rates of Ca^2 +^ entry observed between N- and S-type cells. In S-type cells there was no change in SOCE following differentiation, yet S-type cell populations displayed variable SOCE activity, as judged by the scatter in the relationship between Ca^2 +^ release and Ca^2 +^ entry. It may be that, as multi-potent precursors to several cell types [3,4], S-type cells represent sub-populations with variable SOC compositions. In proliferating N-type cells double bands were detected by STIM1 and TRPC1 antibodies that were not present in differentiated N-type cells. One possibility is that both STIM1 and TRPC1 exist in two states in proliferating N-type cells and in only one state in differentiated N-type cells. The two states detected in proliferating cells could be as a result of covalent modifications, as has been observed with STIM1 [54]. Clearly the functional consequence of any covalent modifications needs to be elucidated.

In summary, our results reveal that the 9*c*RA-induced switch from proliferation to differentiation in N-type cells is accompanied by a remodelling of SOCE that is consistent with changes in the level of expression of STIM1 and Orai1. The finding that SOCE is down-regulated in N-type cells but not in S-type cells strongly suggests that remodelling of Ca^2 +^ signalling is linked to the process of neuronal differentiation. That remodelling of Ca^2 +^ signalling is a feature of the N-type cells could have important implications for neuroblastoma as tumours with a predominantly N-phenotype are more aggressive than tumours with a predominantly S-phenotype [2]. The down-regulation of SOCE in N-type cells may be required to either drive or maintain the switch from proliferation to differentiation, and the roles of STIM1, Orai1 and TRPC1 in this switch remain to be determined. Multiple studies have implicated the involvement of SOCE proteins in proliferation [55–58] and in differentiation [51,59,60]. It is likely that these proteins play a role in the differentiation process itself and may therefore provide a potential therapeutic target in the treatment of neuroblastoma.

## Figures and Tables

**Table 1:** Rates of rise of TG and Ca^2 +^ responses in N- and S-type cells. Rates of rise of TG (200 nM) and Ca^2 +^ (CaCl_2_, 2 mM) were calculated from calibrated traces such as those shown in Fig. 4. Data were log-transformed and 2-way ANOVA was performed to determine the effect of differentiation and the difference between cell types. For N-type cells; proliferating *n* = 19, differentiated *n* = 15. For S-type cells; proliferating *n* = 16, differentiated *n* = 14.

	N-type		S-type	
Rate of rise (μM s^− 1^)	Proliferating	Differentiated	Proliferating	Differentiated
TG	140 ± 0.24	2.00 ± 0.27	2.13 ± 0.29	3.32 ± 0.76
Ca^2 +^	140 ± 0.10	1.66 ± 0.14	3.05 ± 0.41	2.78 ± 0.50
